# Priming, response learning and repetition suppression

**DOI:** 10.1016/j.neuropsychologia.2008.01.018

**Published:** 2008-06

**Authors:** A.J. Horner, R.N. Henson

**Affiliations:** MRC Cognition & Brain Sciences Unit, 15 Chaucer Road, Cambridge CB2 7EF, UK

**Keywords:** Perception, Memory, Fusiform, Prefrontal, fMRI

## Abstract

Prior exposure to a stimulus can facilitate its subsequent identification and classification, a phenomenon called priming. This behavioural facilitation is usually accompanied by a reduction in neural response within specific cortical regions (repetition suppression, RS). Recent research has suggested that both behavioural priming and RS can be largely determined by previously learned stimulus–response associations. According to this view, a direct association forms between the stimulus presented and the response made to it. On a subsequent encounter with the stimulus, this association automatically cues the response, bypassing the various processing stages that were required to select that response during its first presentation. Here we reproduce behavioural evidence for such stimulus–response associations, and show the PFC to be sensitive to such changes. In contrast, RS within ventral temporal regions (such as the fusiform cortex), which are usually associated with perceptual processing, is shown to be robust to response changes. The present study therefore suggests a dissociation between RS within the PFC, which may be sensitive to retrieval of stimulus–response associations, and RS within posterior perceptual regions, which may reflect facilitation of perceptual processing independent of stimulus–response associations.

Repetition priming refers to a change in behavioural response to a stimulus following re-exposure. This change can be expressed in reaction times, accuracy, or response bias, and is often facilitatory in nature. Stimulus repetition has also been associated with a decrease in neural activation within several distinct cortical regions (Schacter & Buckner, 1998), suggesting that exposure to a stimulus can stimulate a form of plasticity capable of altering subsequent neural activity when that stimulus is re-exposured. This potential physiological marker of priming has been termed Repetition Suppression (RS) (Grill-Spector, Henson, & Martin, 2006)

RS is normally found in a number of cortical regions, depending on the nature of the stimulus and the manner in which it is processed (which is normally a function of the experimental task). For example, for tasks involving decisions about familiar visual objects, RS is normally found in higher parts of the ventral visual processing stream and in inferior frontal regions (Koutstaal et al., 2001; Simons, Koutstaal, Prince, Wagner, & Schacter, 2003; van Turennout, Ellmore, & Martin, 2000; Vuilleumier, Henson, Driver, & Dolan, 2002). The former is often attributed to facilitation of perceptual identification, while the latter is often attributed to facilitation of controlled semantic or name retrieval. This common “component-process” view of priming (Henson, 2003) thus assumes that behavioural priming is a consequence of faster or more efficient processing in a number of brain regions that support separate, component processes (Fig. 1A and B).

However, repetition of a stimulus in a specific task may also entail the formation of a more direct association between the stimulus and the response given. These associations can allow the response to be cued by the recurrence of the stimulus, without necessarily requiring repetition of all the processing stages engaged during its prior presentation (Fig. 1C). For example, Logan (1990) proposed a race between these two possible routes to the response – i.e., retrieval of a previous stimulus–response “instance” or reengagement of the “algorithmic” route – with the behavioural response being determined by the faster. Retrieval of the associated response could completely by-pass, or at least curtail, processing within the stages involved in determining the initial response (see also Hommel, 2005).^1^

A recent fMRI study provided compelling evidence for the important role of stimulus–response associations in both priming and RS (Dobbins, Schnyer, Verfaellie, & Schacter, 2004). These authors used a paradigm in which participants judged whether or not the everyday object depicted by a coloured picture was “bigger than a shoebox”. When the stimuli were repeated within this task, in the initial “Start” phase, faster RTs were found (relative to novel stimuli), in conjunction with RS in a number of brain regions, including left prefrontal and fusiform regions. The authors proposed that this RS reflected rapid retrieval of the response previously associated with a repeated stimulus, which bypassed extensive processing in those regions. In support of this proposal, when the task was reversed to a “smaller than a shoebox” judgment, in a subsequent “Switch” phase, both priming and RS for repeated stimuli was reduced. Indeed, RS in the fusiform region was no longer reliable (i.e., apparently abolished). This latter result is surprising, because it suggests that a region normally associated with perceptual processing is no longer affected by repetition of the same attended stimulus. In other words, even a posterior brain region associated with relatively early processing stages can be affected by a response-level manipulation.

One puzzling aspect of these data is that other fMRI studies have found robust RS in fusiform regions under conditions deliberately chosen to limit the occurrence of stimulus–response learning: for example, tasks with no explicit response requirements for the critical stimuli (Henson, Shallice, & Dolan, 2000), changes in the task such that the response on repetition of a stimulus is (on average) orthogonal to its previous response (Henson et al., 2003); or changes in both the stimulus and response, with no obvious stimulus–response pairing, such as in word-stem completion paradigms that involve different tasks at study and test (Schacter, Alpert, Savage, Rauch, & Albert, 1996; Schott et al., 2005). Thus stimulus–response associations would not appear sufficient to explain RS in all brain regions, particularly parts of the ventral visual processing stream.

Another intriguing aspect of Dobbins et al.'s findings concerns the reduced RS when the judgment was reversed. One possible explanation is that, because any responses previously associated with stimuli would no longer help (and indeed possibly hinder), no “bypassing” occurred, and both novel and repeated stimuli underwent the normal “detailed” stage-wise processing. This would imply that neural activity associated with repeated and novel stimuli in the Switch phase should match that for novel stimuli in the “Start” phase. Whether this was the case in the data of Dobbins et al. is unclear. However, even if this were the case, one puzzle is why the prior processing in the relevant regions did not confer any facilitation (“savings”) when stimuli repeated in the Switch phase were re-processed by those regions (given the RS found in the studies discussed above where stimulus–response learning was minimal). One possibility is that the interference induced by retrieval of previous responses required additional or prolonged processing, in order to overcome the prepotent stimulus–response association, and this counteracted any RS in those regions (see Rothermund, Wentura, & De Houwer, 2005; Waszak & Hommel, 2007).

A final issue concerning the Dobbins et al. study is a potential confound when comparing RS for the Start and Switch phases. This concerns the mean lag between initial and repeated presentations of stimuli, which was longer for the Switch phase than for the Start phase (given that the first presentation of repeated stimuli in the Switch phase actually occurred in the previous Start phase). Given that RS normally decreases with increasing lag (Henson, Rylands, Ross, Vuilleumeir, & Rugg, 2004), this factor could explain the reduced RS found in the Switch phase. Note that this potential lag effect could not account fully for the findings of Dobbins et al., because they also found a partial “recovery” of RS in a final “Return” phase, in which the lag was even longer still. Nonetheless, we deemed it important to control for this factor.

The purposes of the present study were therefore to reproduce and extend the findings of Dobbins et al. We replicated the basic paradigm, using the same type of stimuli (a superset of those used by Dobbins et al.) and the same “bigger/smaller than a shoebox” task. However, we switched to a “study-test” design, in which the lag between initial and repeated presentations was matched across the three critical conditions. In the study phases, the task was always “is the object bigger than a shoebox?”, to which participants responded “yes” or “no” using finger presses (Fig. 1D). For the Same condition, this task was repeated at test. For the Reverse condition, the task was switched to “is the object smaller than a shoebox?” (maintaining the mapping of “yes”/“no” to finger press). Finally, we added a third condition – the Orthogonal condition – in which the test task was “is the object man-made?”.^2^ Importantly, the stimuli were selected so that one half of those man-made were bigger than a shoebox, and one half of those not man-made were bigger than a shoebox, so on average the response required for a stimulus at study would not apply to the response required at test. The reason for this third condition was to provide a “baseline” measure of priming/RS, against which any facilitation owing to response repetition (in the Same condition) or any interference owing to response reversal (in the Reverse condition) could be assessed. This condition was also analogous to previous fMRI studies that used classification tasks in which responses were uncorrelated for initial and repeated presentations (Henson et al., 2003).

A final extension was a factorial manipulation of stimulus quality. At test, one half of the stimuli was presented normally but one half was gradually revealed from behind pixel-wise noise (Fig. 1E). The rationale for this stimulus degradation was to increase the perceptual component to priming, given that our previous behavioural research has shown it to be successful in increasing overall priming in this paradigm (Horner & Henson, in preparation). More specifically, we wondered whether, if we, like Dobbins et al., did not observe reliable RS in “perceptual regions” in the Reverse (or Orthogonal) task when using intact and clear stimuli, we might see RS for degraded versions. Given this additional factor, we removed the “low-primed vs. high-primed” factor of Dobbins et al. (in which stimuli were presented either one or three times in the Start phase respectively); rather, all our primed stimuli were only seen once before, as is more typical of behavioural and fMRI studies of priming. The present study therefore used a 3 × 2 × 2 factorial design, with factors “Task” (Same, Reverse, Orthogonal), “Stimulus” (Complete, Degraded) and “Repetition” (Novel, Repeated), for two experiments: Experiment 1 was a behavioural pilot for the main fMRI experiment (Experiment 2).

## 1 Experiment 1—behavioural study

### 1.1 Materials and methods

#### 1.1.1 Participants

Twelve participants (4 male) gave informed consent to participate in the experiment. The mean age across participants was 21.0 years (*σ* = 2.5). All participants were recruited from the MRC-CBU subject panel, or from the student population of Cambridge University. All participants had normal or corrected to normal vision. 2 participants reported as being left-handed, the remaining 10 reported as being right-handed.

#### 1.1.2 Materials

Stimuli were 240 coloured images of everyday objects, largely taken from the set used by Dobbins et al. (2004). They were selected so that 25% were bigger than a shoebox and man-made; 25% were bigger than a shoebox and natural; 25% were smaller than a shoebox and man-made; and 25% were smaller than a shoebox and natural, according to norms taken from independent raters (Horner & Henson, in preparation). Each picture was randomly assigned to one of 12 groups relating to the 12 experimental conditions, with each group containing equal numbers of each stimulus classification, resulting in 20 stimuli per group. The assignment of groups to experimental condition was rotated across participants. The scrambled stimuli used during study blocks (see Section 1.1.3) were created from the same set of objects by randomly re-distributing the pixels so that a coherent object was no longer visible.

#### 1.1.3 Procedure

**Fig. 1:**
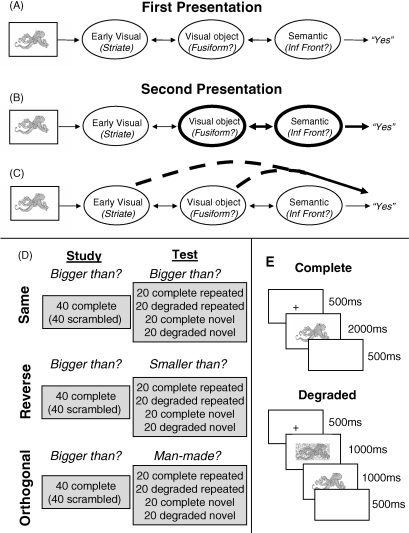
Schematic of hypothetical component processes in a size-judgment task on visual objects. (A) A number of stages are involved in determining the response, the first time a stimulus is presented (shown together with hypothetical associated brain regions). (B) When that stimulus is repeated within the same task, one or more (but not necessarily all) of those stages are facilitated, leading to behavioural priming. (C) Alternatively, a direct response between stimulus and response might be established from the first presentation, which cues the response to a repeated stimulus, effectively bypassing some of the component processes. [Adapted with permission from Henson (in press), The New Encyclopedia of Neuroscience, edited by Larry Squire et al.] (D) Details of the present experimental design, and (E) of the trial sequence at Test. Inf Front = inferior frontal.

The experiment consisted of three study-test cycles, with each cycle lasting approximately 10 min. At Study, stimuli were paired with the question “is it bigger than a shoebox?”, where this comparison referred to the object's typical size in real life. During each Study phase, 80 stimuli were shown, 40 were intact images (which were repeated at test), 40 were scrambled versions of the same stimuli. Complete and scrambled pictures were grouped into mini-blocks of five stimuli, with each mini-block lasting 15 s. During scrambled stimuli mini-blocks, participants were instructed to alternate between right and left key presses at stimulus onset. At Test, stimuli were paired with one of the three test tasks (Fig. 1D). During each Test phase, the 40 stimuli from the Study phase were randomly intermixed with 40 novel stimuli. One half of the items seen at test were complete, the other half degraded (crossed with Novel vs. Repeated). The order of the three test conditions (tasks) was counterbalanced across participants.

An example trial sequence is shown in Fig. 1E. A centrally placed fixation cross was presented for 500 ms, followed by a stimulus for 2000 ms, in turn followed by a blank screen for 500 ms. Participants were able to respond at any point up to the start of a new trial (i.e., the presentation of another fixation cross). For the Degraded trials, the stimulus at onset was completely masked by setting 100% of pixels to gray. The amount of this noise was reduced gradually by randomly removing gray voxels from 100% at onset to 0% after 1000 ms, over 25 steps. The unmasked stimulus then remained on screen for a further 1000 ms.

Participants responded using a “yes” or “no” key with their right or left index finger respectively. Prior to entering the scanner, participants were asked to perform a practice session using the “bigger than a shoebox” task. Although participants were told the question (task) may change during the course of the experiment, the other test tasks were only explained to the subjects prior to a particular test phase.

#### 1.1.4 Behavioural analyses

Accuracy for the shoe-box and man-made tasks was based on prior norms (Horner & Henson, in preparation). Accuracy was close to ceiling, so was not analysed further. Responses with RTs that were two or more standard deviations above or below a participant's mean for a given task, or less than 400 ms, were excluded from analyses. Given the interest in response learning, RTs for Primed items in the Same condition were calculated only from “consistent” trials, where the same “yes” or “no” response was given for that object at both Study and Test (note that this could include trials that were “incorrect” according to the prior norms, but that were likely “correct” according to that participant's idiosyncratic view). Likewise, for the Reverse condition, RTs for Primed items were calculated only for trials in which the response at Study was the opposite of that given at Test. The proportions of trials excluded by this procedure are shown in Table 1. Repetition priming was then calculated as the difference in mean RTs between Novel and Repeated stimuli. All statistical tests had alpha set at .05, and a Greenhouse-Geisser correction was applied to all ANOVAs. *t*-Tests were one-tailed, based on Dobbins et al. (2004), except where stated otherwise.

### 1.2 Results and discussion

The percentages of correct and excluded responses, together with mean RTs, are shown in Table 1. The RTs were entered into a 3 × 2 × 2 (Task × Stimulus × Repetition) repeated-measures ANOVA. There were reliable main effects of task, *F*(1.7, 21.2) = 27.61, *p* < .001, stimulus, *F*(1, 11) = 106.17, *p* < .001, and repetition, *F*(1, 11) = 40.59, *p* < .001. As expected, the main effect of stimulus reflected longer RTs for Degraded than Complete objects. The only significant interaction was between task and repetition, *F*(1.8, 20.3) = 7.32, *p* < .01. This interaction was investigated further, revealing significantly less priming for the Orthogonal condition relative to both the Same, *t*(11) = 4.45, *p* < .01, and Reverse, *t*(11) = 2.10, *p* < .05, conditions. Although the difference between the Same and Reverse conditions did not quite reach significance when collapsing across stimulus, *t*(11) = 1.89, *p* = .09, the decrease in priming for the Reverse condition was significant when the analysis was restricted to Complete objects, *t*(11) = 1.89, *p* < .05, consistent with previous findings (Dobbins et al., 2004; Schnyer et al., 2006).

Given that RTs were generally smaller for Complete than Degraded objects, and smaller for the Orthogonal task relative to the Same or Reverse tasks, analogous analyses were performed using a proportional measure of priming ((novel − repeated)/novel), which makes some allowance for differences in mean RTs (Table 1). A 3 × 2 (Task × Stimulus) ANOVA revealed a main effect of task, *F*(1.9, 21.3) = 8.52, *p* < .01, replicating the reliable Task × Repetition interaction found in the above ANOVA on Novel and Repeated RTs separately (i.e., replicating the results from the standard “additive” measure of priming).

**Fig. 2:**
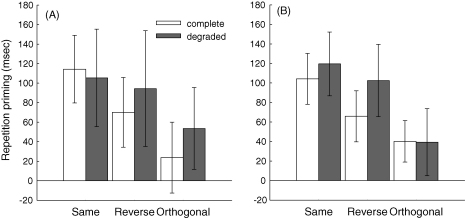
Repetition priming (Novel–Repeated) across Task (Same, Reverse, Orthogonal) and Stimulus (Complete, Degraded) for (A) Experiment 1 and (B) Experiment 2. Error bars represent 95% confidence intervals (one-tailed).

Fig. 2A shows the amount of repetition priming for each Task (Same, Reverse, Orthogonal) and stimulus (Complete, Degraded). One-tailed *t*-tests confirmed reliable priming in every case, *t*(11)s > 1.98, *p*s < .05, except the Orthogonal Complete condition, *t*(11) = 1.28, *p* = .11 (analogous *t*-tests using the proportional priming measure also showed reliable priming in every case, *t*(11)s > 2.50, *p*s < .01, except the orthogonal complete condition, *t*(11) = 1.09, *p* = .14). Consistent with our prediction that stimulus degradation would increase priming (Horner & Henson, in preparation), there was a significant increase in priming for Degraded relative to Complete stimuli in the Orthogonal condition, *t*(11) = 3.76, *p* < .05.

Experiment 1 therefore revealed significant effects of response learning, in that reductions in priming were found for the Reverse relative to Same condition, and for the Orthogonal relative to Same condition. In addition, an increase in priming was found for Degraded relative to Complete objects, at least in the Orthogonal condition, consistent with our previous experiments (Horner & Henson, in preparation) and suggesting a contribution of perceptual (or semantic) facilitation in addition to response learning. We return to these issues in the General Discussion, after considering the data from the fMRI version of the paradigm.

## 2 Experiment 2—fMRI study

### 2.1 Materials and methods

#### 2.1.1 Participants

Eighteen participants (8 male) gave informed consent to participate in the experiment. The mean age across participants was 23.1 years (*σ* = 2.1). All participants were recruited from the MRC-CBU subject panel, or from the student population of Cambridge University. All participants had normal or corrected to normal vision and were right-handed. The study was of the type approved by a local research ethics committee (LREC reference 05/Q0108/401).

#### 2.1.2 Materials and procedure

These were identical to Experiment 1.

#### 2.1.3 fMRI acquisition

Thirty-two T2*-weighted transverse slices (64 × 64 3 mm × 3 mm pixels, TE = 30 ms, flip-angle = 78°) per volume were taken using Echo-Planar Imaging (EPI) on a 3 T TIM Trio system (Siemens, Erlangen, Germany). Slices were 3-mm thick with a 0.75-mm gap, tilted up by approximately 30° at the front to minimise eye-ghosting, and acquired in descending order. Six sessions of 130 volumes were acquired, with a repetition time (TR) of 2000 ms. The first five volumes of each session were discarded to allow for equilibration effects. A T1-weighted structural volume was also acquired for each participant with 1 mm × 1 mm × 1 mm voxels using MPRAGE and GRAPPA parallel imaging (flip-angle = 9°; TE = 2.99 s; acceleration factor = 2).

#### 2.1.4 fMRI analysis

Data were analysed using Statistical Parametric Mapping (SPM5, http://www.fil.ion.ucl.ac.uk/spm5.html). Preprocessing of image volumes included spatial realignment to correct for movement, followed by spatial normalisation to Talairach space, using the linear and nonlinear normalisation parameters estimated from warping each participant's structural image to a T1-weighted template image from the Montreal Neurological Institute (MNI). These re-sampled images (voxel size 3 mm × 3 mm × 3 mm) were smoothed spatially by an 8 mm FWHM Gaussian kernel (final smoothness approximately 11 mm × 11 mm × 11 mm).

Statistical analysis was performed in a two-stage approximation to a Mixed Effects model. In the first stage, neural activity was modelled by a delta function at stimulus onset. The BOLD response was modelled by a convolution of these delta functions by a canonical Haemodynamic Response Function (HRF). The resulting time-courses were down-sampled at the midpoint of each scan to form regressors in a General Linear Model.

For each Test session (Task), nine separate regressors were modelled—the four experimental conditions (Stimulus × Repetition) were split according to the particular key-press given (left/right), plus an additional regressor for discarded trials (using the behavioural exclusion criteria outlined in Experiment 1). To account for (linear) residual artefacts after realignment, the model also included six further regressors representing the movement parameters estimated during realignment. Voxel-wise parameter estimates for these regressors were obtained by maximum-likelihood estimation, using a temporal high-pass filter (cut-off 128 s) to remove low-frequency drifts, and modelling temporal autocorrelation across scans with an AR(1) process.

Images of contrasts of the resulting parameter estimates (collapsed across left/right key-press) comprised the data for a second-stage model, which treated participants as a random effect. In addition to the 18 subject effects, this model had 12 condition effects, corresponding to a 3 × 2 × 2 (Task × Stimulus × Repetition) repeated-measures ANOVA. Within this model, Statistical Parametric Maps (SPMs) were created of the *t* or *F*-statistic for the various ANOVA effects of interest, using a single pooled error estimate for all contrasts, whose nonsphericity was estimated using ReML as described in Friston et al. (2002).

Unless otherwise stated, all SPMs were thresholded at *p* < .05, corrected for multiple comparisons using Random Field Theory, either across the whole-brain or within regions of interest (ROIs) defined by the “main effect” of RS (i.e., using the corrected-thresholded map for the RS T-contrast to define the search volume). Note that defining ROIs by one contrast, such as the main effect, does not bias subsequent contrasts within those ROIs, provided those contrasts are orthogonal, such as the task-by-repetition interaction (Friston, Rotsthein, Geng, Sterzer, & Henson, 2006). Stereotactic coordinates of the maxima within the thresholded SPMs correspond to the MNI template.

#### 2.1.5 Regression analysis

To assess whether the degree of RS across participants was able to predict behavioural priming, a number of regression analyses were performed. First, a single multiple regression was performed to evaluate the relative contribution of three regions of interest (left fusiform, left posterior PFC, left inferior PFC; see Section 3) to the amount of priming across the six conditions (Task × Stimulus). The model therefore included 18 regressors, reflecting RS for each condition and region, plus a further six regressors modelling the mean for each condition. Secondly, a number of additional simple regressions (correlations) of priming were also performed for each region and condition separately (collapsing across Stimulus; see Section 3). Finally, a further simple regression was performed to test whether RS in the Same condition for each region correlated with a behavioural measure of “switch cost”, the difference in each participant's priming between the Same and Reverse tasks, in analogy with Dobbins et al. (2004). To check whether any such correlations were evident outside the ROIs, these simple regressions were repeated over the whole-brain, using corrected thresholds as described above.

## 3 Results

### 3.1 Behavioural results

The percentages of correct and excluded responses, together with mean RTs, are shown in Table 2. The RTs were entered into a 3 × 2 × 2 (Task × Stimulus × Repetition) repeated-measures ANOVA. As in Experiment 1, there were reliable main effects of Task, *F*(1.9, 32.7) = 20.15, *p* < .001, Stimulus, *F*(1, 17) = 279.63, *p* < .001, and Repetition, *F*(1, 17) = 67.74, *p* < .001, and the only significant interaction was between Task and Repetition, *F*(1.9, 34.0) = 10.05, *p* < .001. This interaction reflected less priming for the Orthogonal condition relative to both the Same, *t*(17) = 4.67, *p* < .001, and Reverse, *t*(17) = 2.85, *p* < .01, conditions. Although the difference between the Same and Reverse conditions did not quite reach significance when collapsing Stimulus, *t*(17) = 1.58, *p* = .07, when analysis was restricted to Complete objects the decrease in priming for the Reverse condition was significant, *t*(17) = 2.41, *p* < .05, consistent with Experiment 1 and previous findings (Dobbins et al., 2004; Schnyer et al., 2006). Analysis of the proportional priming measure (Table 2) in a 3 × 2 (Task × Stimulus) ANOVA confirmed a reliable main effect of Task, *F*(1.9, 32.7) = 9.97, *p* < .01, analogous to the Task × Repetition interaction in the above ANOVA on Novel and Repeated RTs separately.

Fig. 2B shows the amount of repetition priming for each Task (Same, Reverse, Orthogonal) and Stimulus (Complete, Degraded). One-tailed *t*-tests confirmed reliable priming in every case, *t*(17) > 1.98, *p* < .05 (which remained when priming was measured proportionally, *t*(17) > 2.00, *p* < .05).

### 3.2 Across-experiments analysis

Though participants performed the Same, Reverse and Orthogonal conditions in different, counterbalanced orders, it is possible that performance on one condition was affected by prior performance of one or more of the other conditions. In order to assess this, the data from Experiments 1 and 2 were combined and subjected to a 3 × 2 × 2 × 6 (Task × Stimulus × Repetition × Order) ANOVA, where the between-subject “Order” factor refers to the six counterbalancing orders of the three conditions. No main effect of Order was present, *F*(5, 24) = .78, *p* = .57, nor did this factor interact significantly with any other factor, *F* < 1.76, *p* > .11.

### 3.3 fMRI results

#### 3.3.1 Main effect of degradation

**Fig. 3:**
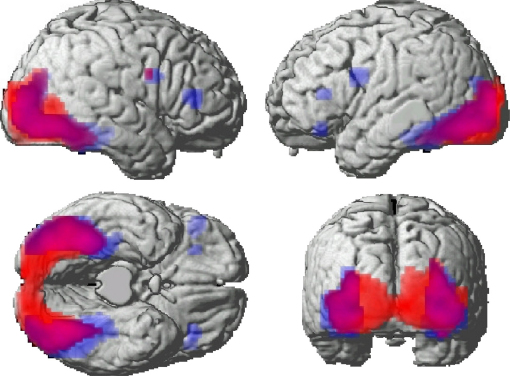
Rendering of activation from the main effect of Degradation (red) and RS (blue). Degradation increased activation within posterior perceptual regions, including the posterior occipital cortex. RS was present within higher-order visual regions, and distinct PFC regions.

This contrast revealed significantly greater activity for Degraded relative to Complete pictures across large bilateral areas of the occipital and posterior inferior temporal lobes, as expected (Fig. 3). A further region within the right posterior prefrontal cortex (+48 +6 +30) also survived correction. Fig. 3 also shows the main effect of RS (see below), and degree of overlap between the effects of degradation and RS. It can be seen that RS was not manifest in the early visual regions showing effects of stimulus degradation (e.g., striate cortex), but did extend into more anterior parts of ventral temporal cortex.

#### 3.3.2 Main effect of repetition

A T-contrast testing for significant RS averaged across all conditions revealed bilateral areas of the lateral occipital and inferior temporal lobes, including the fusiform gyrus, as detailed in Table 3 and Fig. 3. RS in Fusiform has been reported in several previous fMRI experiments utilising similar paradigms (Buckner et al., 1998; Koutstaal et al., 2001; Simons et al., 2003; van Turennout et al., 2000; Vuilleumier et al., 2002). Furthermore, three distinct regions within the prefrontal cortex (PFC) showed RS: bilateral clusters within the posterior PFC, bilateral clusters in the mid-ventrolateral PFC, and a further left inferior PFC region. The left posterior PFC and left fusiform maxima map closely to those focused on by Dobbins et al. (2004) (their Fusiform: −24 −57 −15; their PFC: −45 +6 +27). The only region to show reliable increases in activity for Repeated vs. Novel objects was in precuneus (Table 3).

#### 3.3.3 Repetition × Task interaction

Initial analyses showed no significant interactions between repetition and task that survived correction for whole-brain analysis. Nonetheless, a region in the left inferior PFC (Table 3) showed an interaction that survived small-volume correction for the (orthogonal) main effect of RS.

Neither further interactions nor the main effect of Task reached significance in either the corrected whole-brain analysis, or using a small-volume correction for those voxels that showed significant RS.

#### 3.3.4 Regions of interest

**Fig. 4:**
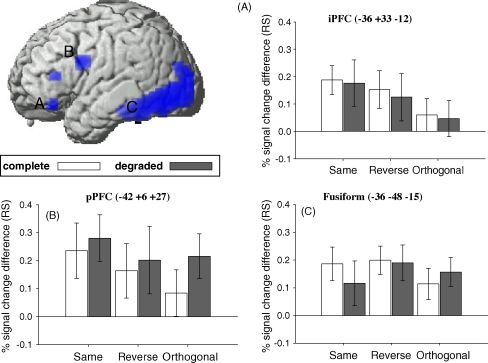
RS for each Task (Same, Reverse, Orthogonal) and Stimulus (Complete, Degraded) in (A) Left inferior PFC; (B) left posterior PFC; (C) left fusiform cortex. Percentage signal change refers to the peak of the fitted BOLD impulse response, and is relative to the grand mean over all voxels and scans. Error bars represent 95% confidence intervals (one-tailed).

Three regions of interest (ROIs) were identified for further analyses: (1) the left fusiform cortex (−36 −48 −15); (2) the left posterior PFC (−42 +6 +27); (3) the left inferior PFC (−36 +33 −12). Regions (1) and (2) were chosen because they were close to the maxima examined in detail by Dobbins et al. (2004). Region (3) was not reported by Dobbins et al., but was the only region to show a reliable interaction between Task and Repetition in the present data (see above). Data for each ROI were extracted from the peak voxel identified for the main effect of RS; the resulting magnitude of RS across each condition (and the effects of degradation) are shown for each ROI in Fig. 4 (the data for each condition separately are shown in Table 4). (Homologous regions in the right-hemisphere showed essentially the same patterns.)

These data were entered into a 3 × 2 (Task × Stimulus) ANOVA on repetition effects. The only effect to reach significance was within the inferior PFC, which showed a main effect of task, *F*(1.8, 31.4) = 6.45, *p* < .01, reflecting greatest RS for the Same and least for the Orthogonal condition (Fig. 4A). This result reproduces the Task-by-Repetition interaction in this region found in the whole-brain analysis, and was further confirmed by pairwise *t*-tests across conditions, which showed significantly less RS for the Orthogonal compared to both the Same, *t*(17) = 5.51, *p* < .001, and Reverse, *t*(17) = 2.12, *p* < .05, conditions (any difference between the Same and Reverse condition failed to reach significance, *t*(17) = 1.14, *p* = .13).

Although no main effect of task was present within the posterior PFC, *F*(2.0, 33.7) = 1.85, *p* = .17, the numerical pattern was similar to that in the inferior PFC region, with greatest RS in the Same condition and least in the Orthogonal condition (Fig. 4B). Two further pairwise tests were performed. First, RS in the Same vs. Reverse conditions was compared for Complete objects only, since this is the contrast closest to that tested by Dobbins et al. (2004). However, any evidence for reduced RS in the Reverse condition was marginal, *t*(17) = 1.02, *p* = .08. The second pairwise test contrasted the Same and Orthogonal conditions, collapsing across Stimulus level. This revealed reliable evidence for smaller RS in the Orthogonal condition, *t*(17) = 1.98, *p* < .05.

In the Fusiform ROI, there was no trend for decreases in RS across the Same to Reverse to Orthogonal tasks (Fig. 4C), unlike in the prefrontal ROIs. Furthermore, RS was significant in every condition. The only ANOVA effects to reach significance were the main effects of Stimulus, *F*(1, 17) = 21.7, *p* < .001, and of repetition, *F*(1, 17) = 72.9, *p* < .001. The main effect of Stimulus reflected greater activity for Degraded than Complete objects, as expected (Table 4).

In order to get stronger evidence for a dissociation between PFC and Fusiform, a 3 × 2 × 2 (Task × Stimulus × Region) ANOVA on the data from inferior PFC and Fusiform regions was performed, which revealed a significant Task × Region interaction, *F*(1.9, 64.6) = 3.38, *p* < .05. The significant main effect of Task present in the inferior PFC was therefore significantly greater than the non-significant Task effect in the Fusiform.

#### 3.3.5 Correlations with behaviour

Dobbins et al. (2004) also reported some intriguing correlations across participants between the amount of RS in their ROIs and the size of various behavioural effects. We first performed a multiple regression, in which RS for each participant in all three ROIs across the six (Task × Stimulus) conditions were used as regressors to predict each participant's behavioural priming (see Sections 1.1 and 2.1). This showed a significant positive relationship between RS and priming, *t*(84) = 1.85, *p* < .05, when collapsing across all tasks and regions, though no evidence for a modulation in this relationship by Task, *F*(2, 84) = 1.00, *p* = .37, ROI, *F*(2, 84) = .07, *p* = .93, or Stimulus, *F*(2, 84) = 1.20, *p* = .12.

**Fig. 5:**
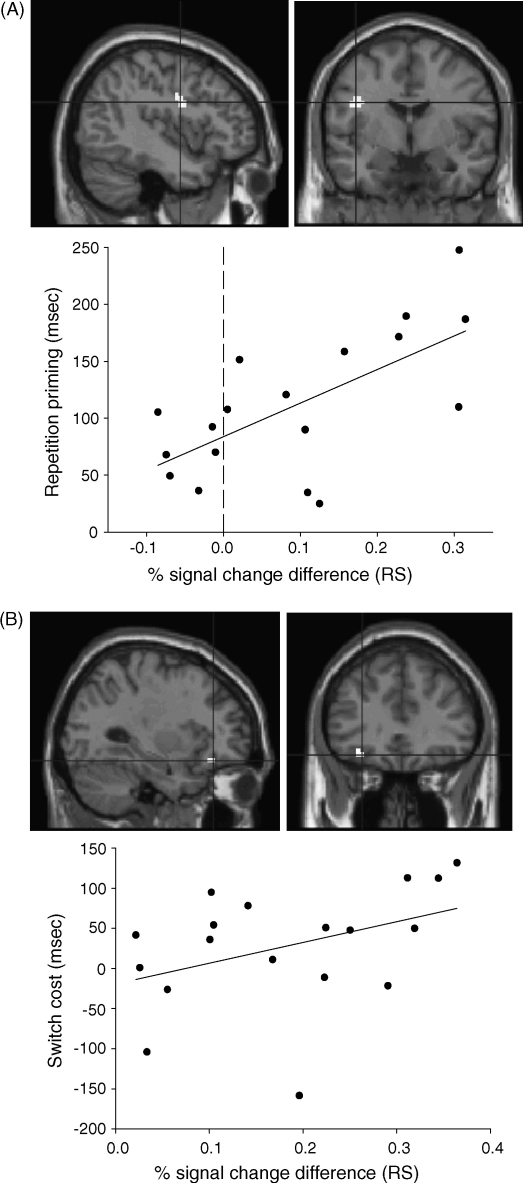
Correlations between behavioural RTs and RS in the Same task within the PFC. (A) RS against repetition priming within the posterior PFC; (B) RS against switch cost within the inferior PFC.

To investigate individual ROIs in more detail, given Dobbins et al.'s findings, simple regressions were also performed for each ROI and Task separately (collapsed across Stimulus). Only posterior and inferior PFC regions showed reliable positive correlations with priming, in both the Same and Orthogonal conditions (Table 5). Finally, in analogy with Dobbins et al. (2004) (see Sections 1.1 and 2.1), simple regression was performed for each region on the behavioural “switch cost” between the Same and Reverse conditions against RS in the Same condition. In this case, only the inferior PFC showed a reliable positive correlation (Table 5 and Fig. 5; though we note the switch cost correlation in the fusiform approached significance, *p* = .08).

While there is clearly a multiple comparison issue with the number of correlations performed in Table 5, it is noteworthy that the significant correlation between posterior PFC and priming in the Same condition replicates the findings of Dobbins et al. (2004): that is, participants who exhibited greater RS in left posterior PFC showed greater priming (see also Bunzeck, Schutze, & Duzel, 2006; Maccotta & Buckner, 2004; Orfanidou, Marslen-Wilson, & Davis, 2006). This positive correlation would appear to apply to the present inferior PFC region too. Furthermore, the inferior PFC region showed a correlation between RS and switch costs: that is, participants who exhibited greater RS in the Same condition also showed greater behavioural “interference” (i.e., a greater reduction in priming) when the task was reversed. A similar result was found by Dobbins et al. (2004), though in their posterior PFC region rather than the present inferior PFC region.

To check whether any brain regions other than the three ROIs showed correlations with behavioural priming or switch costs, analogous regression analyses were performed for every voxel in an SPM analysis. At corrected thresholds, the only additional region to show a correlation was a right posterior PFC region (+39 +9 +27), homologous to left posterior PFC ROI, which correlated with priming in the Same condition.

## 4 General discussion

The two main findings of the present study were that: (1) task changes modulated the amount of behavioural priming and the degree of RS in parts of the PFC, consistent with Dobbins et al. (2004), although a more anterior inferior region of left PFC showed a clearer modulation than did the posterior left PFC region previously reported and (2) task changes did not reliably modulate RS in the fusiform cortex, which showed reliable RS across all tasks, contrary to Dobbins et al. (2004). These findings reinforce Dobbins et al.'s claim that stimulus–response associations play an important role in priming and in RS, at least in paradigms like the present one, but additionally suggest that further factors, such as perceptual facilitation for example, also contribute. We expand on these points below.

### 4.1 Stimulus–response associations

Behaviourally, switching the task from “bigger than a shoebox?” at study to “man-made?” at test, or reversing it to “smaller than a shoebox?” at test, reduced the amount of priming relative to repeating the same task at study and test (in both Experiment 1 and Experiment 2). This reduction may reflect a strategic change from the retrieval of previous responses (in the Same condition), reverting to the use of a slower “algorithmic” processing route (in the Reverse and Orthogonal conditions). This strategic change is likely to be enabled by the fact that participants were aware of the irrelevancy of previously learned responses in the Orthogonal and Reverse conditions. The fact that these effects were found after only one study presentation (corresponding to the low-primed condition in Dobbins et al., 2004) suggests that such stimulus–response associations can be formed quickly, and apply even to conventional “one-shot learning” priming paradigms. Nonetheless, the reliable priming that remained in both the Orthogonal task and the Reverse task suggest that stimulus–response associations cannot explain all priming.

The finding that priming was greater in the Reverse condition than the Orthogonal condition suggests that response learning does not occur solely at the level of a specific finger press, or even at the level of a specific decision, i.e., “yes” or “no”. This is because retrieval of such responses would help on one half of trials in the Orthogonal condition, but none of the trials in the Reverse condition. Rather, the response associated with stimuli may be at the level of a size-judgement decision, e.g. “bigger than”. Retrieval of this decision would help in the Reverse condition, despite a switch in yes/no response, but would be irrelevant in the Orthogonal condition (see Horner & Henson, in preparation, for further data and discussion concerning multiple levels of response representation, and the role of semantic overlap).

Three aspects of the data implicate PFC in mediating effects of such stimulus–response associations. Firstly, RS was significantly modulated by Task (at least when comparing Same vs. Orthogonal conditions), in both the posterior and inferior left PFC. Secondly, priming correlated with RS in both posterior and inferior PFC for the Same condition. Thirdly, RS in the left inferior PFC during the Same task correlated with participants’ behavioural switch cost between Same and Reverse tasks. Importantly, none of these effects were found in fusiform cortex, despite robust RS in this region. This general dissociation between RS in PFC and RS in posterior temporal/occipital regions is consistent with data from TMS on PFC during the initial exposure to objects (Wig, Grafton, Demos, & Kelley, 2005).

### 4.2 PFC and semantic retrieval vs. response retrieval

Left posterior PFC showed reliable RS in all conditions, consistent with Dobbins et al. (2004). Contrary to the results of Dobbins et al., no reliable reduction in RS was seen in the Reverse task (relative to the Same task); however, a significant reduction was seen with the inclusion of our Orthogonal task. A similar pattern across the three tasks was seen in the left inferior PFC (though here, RS was no longer reliable in the Orthogonal condition). This pattern of greater RS in the Same and Reverse tasks than in the Orthogonal task is consistent with the hypothesised role of ventrolateral PFC (VLPFC) in semantic retrieval (Wagner, Pare-Blagoev, Clark, & Poldrack, 2001), since retrieval of size information was relevant to both the Same and Reverse task, but not the present Orthogonal task. However, the additional correlation between RS in the inferior PFC region and the switch cost across Same and Reverse tasks would appear difficult to explain purely in terms of semantic retrieval. This finding (like that of Dobbins et al., 2004) would appear more easily explained in terms of stimulus–response associations. More specifically, if RS in PFC reflects such response learning, then participants who show evidence of greater response-learning (as indexed by RS in the Same task) should show a greater switch cost, i.e., less priming in the Reverse task, where a previously learned association has to be ignored in order to answer correctly.

This alternative account of activity in inferior PFC resembles the hypothesised role of mid-VLPFC in post-retrieval selection of information retrieved from semantic memory (Badre & Wagner, 2007), though we note that the present inferior PFC is more medial and anterior than the mid-VLPFC defined by Badre & Wagner (2007). More generally, the pattern of RS across the present inferior vs. posterior PFC regions is consistent with Badre and Wagner's hypothesis that VLPFC processing is organised hierarchically along a rostro-caudal axis en route to action. In this conception, left inferior PFC is sensitive to changes in both semantic overlap and response-learning associated with task switches, whereas the left posterior PFC, due to its proximity to premotor cortex, may be more tightly connected to RT measures of priming regardless of task switches. We note, however, that two results are difficult to fit within this conception: (1) the significant correlation between RS and priming in the Orthogonal condition within the inferior PFC; (2) the lack of significant correlation between RS and priming in the Reverse condition within the posterior PFC. Future studies of response-learning may find more clear-cut functional dissociations between regions within PFC.

### 4.3 Fusiform and perceptual facilitation

RS in fusiform cortex was not reliably modulated by task; indeed it was reliable in every condition, contrary to Dobbins et al. (2004), and including the Orthogonal condition, consistent with previous studies (e.g., Henson et al., 2003). One explanation is that facilitation of perceptual processing occurs whenever a stimulus is repeated and the task entails processing at least to the level of object identification.^3^ This could explain the residual behavioural priming found even in the Orthogonal task where effects of response learning may be less prevalent. It is also consistent with previous demonstrations of RS in ventral temporal regions during paradigms where responses at study (e.g., pleasantness ratings on words) are completely unrelated to those at test (e.g., completing a word from three initial letters; Thiel, Henson, Morris, Friston, & Dolan, 2001). One puzzle concerning this explanation however is why no reliable correlation was found between RS in fusiform and the amount of priming across participants. This lack of correlation has been noted previously (Bunzeck et al., 2006; Maccotta & Buckner, 2004; Sayres & Grill-Spector, 2006; Xu, Turk-Browne, & Chun, 2007).^4^

One reason could be that the binary semantic classification tasks performed on clear images of everyday objects, like that used here and in many previous studies, load more heavily on post-perceptual processing. In other words, perceptual components may contribute only a small portion of the total RT variance in such tasks. If so, the lack of correlation could simply reflect low power to detect a small effect size in RTs (given large variability from other, post-perceptual sources). Alternatively, some of the variance in behavioural RTs may not correlate with mean activity in individual regions, but rather with repetition-induced changes in the effective connectivity between regions.

As expected, visually degrading the test stimuli increased overall activity for novel and repeated stimuli within early visual cortex. In previous experiments (Horner & Henson, in preparation), we found that such degradation increases overall priming (see also Waszak & Hommel, 2007), as might be expected if faster identification of degraded stimuli, owing to prior exposure to a complete version, boosted the relative contribution of the perceptual component to RTs. We replicated this in the Orthogonal condition of Experiment 1, though it did not reach significance in Experiment 2. Surprisingly however, this manipulation did not result in an increase in the amount of RS in fusiform cortex (or any region): i.e., degradation increased overall fusiform activity for novel and primed (Table 4), but not the size of the difference in activity between them. This manipulation of visual degradation therefore failed to provide further support for the attribution of fusiform RS to facilitation of perceptual processing.^5^

An alternative explanation is that fusiform RS reflects processing largely irrelevant to the behavioural measure of priming in paradigms like the present one. For example, the RS in fusiform may reflect processes arising subsequent to the behavioural response, such as reductions in attention to the object once a decision has been made (see also Eger, Henson, Driver, & Dolan, 2007). This possibility raises other questions however, like how to explain why lesions to posterior brain regions can cause a deficit in priming (Gabrieli, Fleischman, Keane, Reminger, & Morrell, 1995), or why TMS on nearby perceptual regions (lateral occipital cortex) can modulate priming (Pobric, Schweinberger, & Lavidor, 2007).

### 4.4 Differences from Dobbins et al. (2004)

Given some discrepancies between the present data and those of Dobbins et al. (2004), some of the procedural differences between the two investigations deserve consideration. One reason why task reversal did not have such large effects on RS in the present fMRI data may be that response learning played a lesser role in the present study. One obvious difference is that we only employed what Dobbins et al. called “low-primed” items (presented only once before the task switch), and there seems little doubt that multiple repetitions should increase the relative impact of response learning (Logan, 1988). However, some of the key fMRI results of Dobbins et al., e.g., lack of RS in fusiform, were found even for low-primed items (though it remains possible that the mere presence of high-primed items at study encourages greater utilisation of stimulus–response learning). It is noteworthy in this context that mean RTs in the present study were faster than those in the Dobbins et al. (2004) study (Same Complete Novel: 879 ms vs. 940 ms respectively; Reverse Complete Novel: 938 ms vs. 980 ms respectively). If repetition priming does involve a “race” between an algorithmic process and automatic stimulus–response associations (Logan, 1990), faster RTs for novel items may lower the probability of stimulus–response associations winning the “race” (Waszak & Hommel, 2007).

Another procedural difference was the present intermixing of degraded stimuli at test. As discussed above, stimulus degradation has previously been shown to increase priming, irrespective of task switches, when used as a between-subjects factor (Horner & Henson, in preparation; Waszak & Hommel, 2007). The inclusion of both degraded and complete stimuli within the same test block however may have disrupted response-learning effects. For example, the visual discrepancy between (complete) stimuli at study and degraded stimuli at test may have ameliorated cueing of responses in the Degraded condition. This may even have altered participants’ overall strategy, limiting the contribution of stimulus–response associations in all conditions. These possibilities require further investigation.

Finally, we note that repeated stimuli produced greater activity than novel stimuli in the Precuneus. This region has previously been implicated in episodic retrieval (Wagner, Shannon, Kahn, & Buckner, 2005). There is little doubt that participants in the present study were conscious of the repetition of objects. It is possible that conscious recollection of their response at study contributed to priming (despite instructions to respond as quickly as possible). Indeed, it is possible that stimulus–response associations (at least in the present type of paradigm) operate at the level of conscious retrieval. It is noteworthy in this respect that amnesic patients show impaired stimulus–response learning (Schnyer et al., 2006).

## 5 Conclusions

The present data provide evidence for a dissociation between RS in the PFC, which is significantly modulated by task changes, and RS in the fusiform cortex, which appears resilient to changes in task. RS in the PFC seems to be related to some form of stimulus–response learning. RS in the PFC also plays a clear role in behavioural priming, whereas the role of the fusiform is unclear. It should be noted however that these conclusions may be a consequence of the specific paradigm used here (and by others), in which perceptual demands are low (at least for complete objects) and semantic and/or executive demands are high (given the rather ad hoc classification, viz. object size relative to a shoebox). Paradigms with greater perceptual demands (e.g., object-fragments) and fewer semantic/executive demands (e.g., naming, or more basic-level categorisations) may reveal a stronger correlation between fusiform RS and behavioural measures of priming, and less influence of stimulus–response learning.

## Figures and Tables

**Table 1:** Mean percentage accuracy (acc.), mean percentage excluded trials (exc., see Sections 1.1 and 2.1), and reaction times (RTs), together with proportional priming (prop. pri.) across Task, Stimulus and Repetition for Experiment 1

	Same		Reverse		Orthogonal	
	Complete	Degraded	Complete	Degraded	Complete	Degraded
Acc.						
Novel	84.6 (7.2)	87.9 (6.9)	86.3 (3.8)	82.1 (7.8)	96.7 (3.9)	96.3 (5.3)
Primed	89.6 (5.0)	88.8 (7.4)	85.8 (7.3)	83.8 (10.0)	95.0 (4.3)	97.9 (2.6)

Exc.						
Novel	2.9 (5.7)	11.4 (3.8)	2.9 (3.9)	9.3 (6.7)	1.4 (2.4)	12.1 (4.8)
Primed	7.9 (5.7)	15.0 (9.1)	14.3 (8.4)	15.0 (5.8)	1.4 (2.4)	5.7 (3.5)

RTs						
Novel	889 (124)	1112 (113)	1037 (181)	1313 (197)	781 (118)	1053 (115)
Primed	774 (116)	1007 (85)	967 (162)	1218 (207)	757 (82)	999 (135)

Prop. Pri.	.13 (.06)	.09 (.08)	.06 (.06)	.07 (.08)	.02 (.07)	.05 (.06)

S.D. within parentheses.

**Table 2:** Mean percentage accuracy (acc.), mean percentage excluded trials (exc., see Sections 1.1 and 2.1), and reaction times (RTs), together with proportional priming (prop. pri.) across Task, Stimulus and Repetition for Experiment 2

	Same		Reverse		Orthogonal	
	Complete	Degraded	Complete	Degraded	Complete	Degraded
Acc.						
Novel	85.3 (7.2)	86.1 (8.8)	81.7 (11.4)	85.8 (6.9)	96.7 (3.8)	96.9 (3.9)
Primed	87.8 (8.6)	85.8 (7.7)	83.3 (7.7)	87.2 (8.3)	97.5 (3.0)	94.4 (3.4)

Exc.						
Novel	0.8 (2.6)	14.2 (8.1)	1.1 (2.1)	9.7 (6.7)	0.8 (1.9)	8.9 (4.4)
Primed	11.4 (8.2)	14.4 (11.1)	15.6 (7.8)	14.8 (9.0)	0.8 (1.9)	7.5 (5.8)

RTs						
Novel	879 (113)	1178 (180)	938 (127)	1249 (147)	792 (140)	1053 (176)
Primed	775 (104)	1058 (124)	872 (110)	1146 (170)	752 (120)	1014 (153)

Prop. Pri.	.12 (.06)	.10 (.06)	.07 (.07)	.08 (.06)	.05 (.02)	.03 (.07)

S.D. within parentheses.

**Table 3:** Regions showing effects of repetition suppression (Novel > Repeated) and repetition enhancement (Repeated > Novel), *p* < .05 whole-brain corrected, five voxel extent threshold; Regions showing Repetition-by-Task interaction, *p* < .05 SVC for main effect of repetition suppression

Region	Voxels	MNI co-ordinates			*Z*-score
Novel > Repeated					
Fusiform gyrus	933/859	+36/−36	−48	−15	>7.84
Posterior PFC	52/38	+42/−42	+6	+27	6.62/6.12
Mid-lateral PFC	57/11	+48/−45	+36/+30	+12	6.32/5.21
Left Inferior PFC	24	−36	+33	−12	5.72

Repeated > Novel					
Precuneus	412	+15	−63	+33	7.75

Repetition × Task					
Left Inferior PFC	23	−30	+33	−15	3.63

**Table 4:** Mean percent signal change (and S.D.) within the Fusiform (fusi.), posterior PFC (pPFC) and inferior PFC (iPFC) across Task, Stimulus and Novel vs. Primed

Region	Same		Reverse		Orthogonal	
	Complete	Degraded	Complete	Degraded	Complete	Degraded
Fusi.						
Novel	.43 (.35)	.50 (.40)	.40 (.31)	.45 (.36)	.25 (.31)	.40 (.31)
Primed	.25 (.30)	.39 (.40)	.20 (.29)	.26 (.31)	.14 (.29)	.25 (.31)

pPFC						
Novel	.11 (.66)	.22 (.66)	.14 (.58)	.20 (.62)	.14 (.44)	.32 (.55)
Primed	−.13 (.58)	−.06 (.58)	−.02 (.57)	.00 (.57)	.05 (.49)	.10 (.49)

iPFC						
Novel	.14 (.34)	.17 (.27)	.14 (.36)	.06 (.46)	.13 (.38)	.16 (.34)
Primed	−.05 (.35)	−.01 (.35)	−.02 (.43)	−.06 (.47)	.07 (.35)	.11 (.37)

Percent signal change refers to the peak of the fitted BOLD impulse response, and is relative to the grand mean over all voxels and scans. Note that the baseline level of 0 was not estimated reliably in this design, so only relative patterns across conditions are meaningful.

**Table 5:** Results of simple regressions of behavioural priming and switch cost against RS within three regions: left fusiform, left posterior PFC (pPFC), left inferior PFC (iPFC), collapsed across Stimulus (Complete/Degraded)

Dependent variable	Region		
	Fusiform	pPFC	iPFC
Priming same	+0.2	+0.42*	+0.42*
Priming reverse	−0.24	+0.36	+0.14
Priming orthogonal	+0.26	+0.47*	+0.56*
Switch cost (same–reverse)	+0.36	+0.14	+0.40*

Values represent Pearson's *R* (*p < .05).

## Footnotes

1 Note that the “response” is not necessarily at the level of the motor command (e.g., specific finger press), but can be at a more abstract level, e.g., the decision made in a binary classification task (what Logan, 1990, called the stimulus “interpretation”)—see also Schnyer, Dobbins, Nicholls, Schacter, and Verfaellie (2006), Horner and Henson (in preparation). Note also that response retrieval may require some level of successful stimulus recognition too, suggesting that perceptual contributions may independently affect both priming and RS, though it is presently unclear at what level such recognition occurs.

2 A reviewer noted that one potential confound of this design is that effects of task-switching may reflect differences in the specific task used at test (e.g., if the man-made task is “easier” than the shoe-box task, then the amount of priming may be disproportionately affected). An alternative design would keep the Test task constant, but vary the Study tasks accordingly (though potential task differences might then arise in the “encoding” of study trials instead).

3 Though we note that posterior fusiform activity has also been attributed to semantic processing (e.g., Wheatley, Weisberg, Beauchamp, & Martin, 2005).

4 Dobbins et al. (2004) actually found a negative correlation between fusiform RS and priming in their Start phase (though not their Return phase, which may reflect its longer lag between initial and repeated presentations).

5 We note that a previous fMRI experiment showed repetition enhancement, rather than RS, when using visually degraded stimuli (Turk-Browne, Yi, Leber, & Chun, 2007). The reason for this apparent discrepancy with our findings is most likely because our degraded stimuli were still invariably identified (given that the degradation eventually disappeared, leaving a complete object shown for 1000 ms), whereas the degraded stimuli used by Turk-Browne et al. were only identified on approximately 70% of trials. When priming leads to an increase in identification rates, it has been hypothesised that repetition enhancement results (Henson, 2003), because the activation of object-responsive regions that is normally associated with successful identification will occur more often for primed than unprimed trials. In the present paradigm therefore, where identification occurs on all trials, primed and unprimed, there is no reason for repetition enhancement. Rather, the faster identification of objects when gradually revealed from behind our noise masks is likely to cause repetition suppression.
